# Microbial Community Shifts during Biogas Production from Biowaste and/or Propionate

**DOI:** 10.3390/bioengineering2010035

**Published:** 2015-02-09

**Authors:** Chaoran Li, Christoph Moertelmaier, Josef Winter, Claudia Gallert

**Affiliations:** 1 Institute of Biology for Engineers and Biotechnology of Wastewater, Karlsruhe Institute of Technology (KIT), Am Fasanengarten, Karlsruhe D-76128, Germany; E-Mails: chaoran.li3@kit.edu (C.L.); christoph.moertelmaier@kit.edu (C.M.); josef.winter@kit.edu (J.W.); 2 Division Microbiology-Biotechnology, Faculty of Technology, University of Applied Science, Hochschule Emden-Leer, Constantiaplatz 4, Emden D-26723, Germany

**Keywords:** anaerobic co-digestion, biowaste, propionate, propionate-oxidizing bacteria (POB), methanogenic community, community shifts

## Abstract

Propionate is the most delicate intermediate during anaerobic digestion as its degradation is thermodynamically unfavorable. To determine its maximum possible degradation rates during anaerobic digestion, a reactor was fed Monday to Friday with an organic loading rate (OLR) of 12/14 kg COD_biowaste_·m^−3^·d^−1^ plus propionate up to a final OLR of 18 kg COD·m^−3^·d^−1^. No feed was supplied on weekends as it was the case in full-scale. To maintain permanently high propionate oxidizing activity (POA), a basic OLR of 3 kg COD_propionate_·m^−3^·d^−1^ all week + 11 kg COD_biowaste_·m^−3^·d^−1^ from Monday to Friday was supplied. Finally a reactor was operated with an OLR of 12 kg COD_biowaste_·m^−3^·d^−1^ from Monday to Friday and 5 kg COD_propionate_·m^−3^·d^−1^ from Friday night to Monday morning to maintain a constant gas production for permanent operation of a gas engine. The propionate degradation rates (PDRs) were determined for biowaste + propionate feeding. Decreasing PDRs during starvation were analyzed. The POA was higher after propionate supply than after biowaste feeding and decreased faster during starvation of a propionate-fed rather than a biowaste-fed inoculum. Shifts of the propionate-oxidizing and methanogenic community were determined.

## Bullet Points

Anaerobic digestion of biowaste + propionate
Periodic biowaste replacement by propionate
Propionate-oxidizing bacteria and methanogenic archaea
Constant biogas production with different substrates
Community changes during different operational conditions

## 1. Introduction

Propionate is a key intermediate of anaerobic digestion in general and of biowaste in particular [1,2,3,4,5,6,7]. Main sources of propionate in bioreactors are odd numbered fatty acids from lipolysis of fat and oil as well as from carbohydrate and amino acid degradation [3]. Propionate accumulates during disturbances of the anaerobic digestion process, caused by e.g., toxic substances, high dry matter content, or high OLR close to overload, when hydrolysis, acidogenesis, acetogenesis, and methanogenesis, leading to biogas formation from complex organic matter, are no longer balanced [4,7,8]. Degradation of accumulated fatty acids such as propionate, however, is thermodynamically unfavorable [9]. Only a few slow-growing and obligately syntrophic propionate-oxidizing bacteria (POB) can degrade propionate to acetate, CO_2_, and hydrogen in the absence of electron acceptors such as sulfate. Degradation of propionate under strictly anaerobic conditions requires a hydrogen partial pressure of <6.5 Pa in a narrow thermodynamically defined window. Thus, in many anaerobic digesters high propionate concentrations can persist for a long time without failure of methane production, e.g., after start up or at high load conditions [1,4,7]. *Deltaproteobacteria* of the genera *Syntrophobacter* and *Smithella*, as well as *Firmicutes* belonging to the genera *Pelotomaculum* and *Desulfotomaculum*, are known to degrade propionate [5]. The growth rate of these POB and their community density in anaerobic digesters is, however, low*.* After a specific enrichment of methanogens for removal of hydrogen, doubling times of a defined methanogenic co-culture of *Pelotomaculum schinkii* of 1.5 days was achievable [10]. For fast propionate degradation, it is essential that acetogenic and methanogenic microorganisms have close spatial proximity to enable an optimal interspecies electron transfer [11].

As *Pelotomaculum* sp. [10] were the latest described genus of POB, earlier publications on propionate degradation did not cover them, although they may have been present. More recent studies revealed that *Pelotomaculum* sp. might represent the dominant POB group in anaerobic digestion systems. Shigematsu* et al.* [12], for instance, analyzed the community structure of POB in a propionate-fed chemostat at different dilution rates and found that at a dilution rate of 0.3 d^−1^ *Pelotomaculum* was the most numerous genus within the POB. Ban* et al.* [13] used qPCR to determine POB in a UASB reactor fed with propionate and proved that *Pelotomaculum schinkii* was the dominating species of POB. Ariesyady* et al.* [14] found high proportions of *Smithella* sp. and lower proportions of *Syntrophobacter* sp. in anaerobic sludge, but they did not determine *Pelotomaculum* sp. In a very recent work by Li* et al.* [15] *Pelotomaculum* sp. comprised 1.2% of the total prokaryotes in manure-straw co-digestion experiments. The same authors observed a positive correlation between the proportion of *Pelotomaculum* sp. and *Methanoculleus* sp., which was already reported by Shigematsu* et al.* [12]. *Methanoculleus* sp. apparently represented not only the optimal methanogenic partners for syntrophic propionate oxidation but also for syntrophic acetate oxidation, as discussed by Hori* et al.* [16].

Only a few studies exist in which the composition of POB in biowaste digesters was analyzed under different process conditions. McMahon* et al.* [1], for instance, observed that POB subgroups changed during start-up or at near-overload phases: Whereas the number of *Smithella* sp. declined after overload, the number of *Syntrophobacter* sp. remained almost unchanged. At steady state conditions the percentage of the POB (not including *Pelotomaculum* sp.) was 1.5–1.6% of total prokaryotes. When in a full-scale biowaste digester in addition to *Syntrophobacter* sp. and *Smithella* sp., *Pelotomaculum* sp. were also analyzed, the proportion of POB within the total prokaryotes increased to 2.7–5% [7].

Fermentation of source-sorted biowaste in continuously operated anaerobic reactors at constant OLR clearly below overload should be attempted. However, due to biowaste collection only on working days, sufficient biowaste suspension may not be available on weekends or holidays. Liquid-acidified substrates from canteens might be used as substitutes for biowaste, but experience with periodic short-term partial replacement of biowaste or a complete substitution of biowaste with easy-to-handle liquid food residues that could be added over weekends without the requirement of human control is not available. In pre-acidified substrates, propionate is the most sensitive intermediate that requires a high activity of POB. A detailed knowledge of the behavior of POB and the propionate metabolism during biowaste co-digestion with propionate or alternating biowaste-propionate digestion (as a model for acidified food wastes) and the long-term behavior is missing. Thus, in this contribution a suspension of source-sorted biowaste ± little “background-propionate” (that might originate from acidification before collection or was externally added to maintain active POB) was digested with increasing amounts of propionate as a model-substrate for pre-acidified wastewater in a fed-batch digester for 5 days per week without feeding on Saturdays and Sundays, to determine maximal propionate degradation activity. Another reactor was fed with only biowaste from Monday to Friday and only propionate over the weekend to check whether an abrupt substrate change from complex biowaste to propionic acid could maintain a stable biogas production. Propionate degradation rates and community shifts during biowaste and/or propionate digestion should be determined. The stability of propionate oxidation after biowaste and/or propionate feeding with length of starvation should also be investigated and changes of POB and methanogens during the experiments should be analyzed by fluorescence *in situ* hybridization.

## 2. Materials and Methods

### 2.1. Source of Fresh Biowaste and Digester Residues for Inoculation of Laboratory Reactors

One portion of source-sorted biowaste was mixed with two portions of process water for disruption of fibers in an 18-m^3^ hydropulper. The suspension was stored in an interim 30-m^3^ storage tank for subsequent wet anaerobic digestion in the biowaste digestion plant of Karlsruhe, Germany [2,17]. For laboratory experiments from the storage tank, 100 L of biowaste suspension were collected initially and another 100 L 55 days later (for composition, see Table 1). The suspension was frozen in 2-L portions until use. For digestion of biowaste + propionate, respective amounts of concentrated propionic acid were mixed into the daily portion of fresh biowaste suspension, whereas for determining propionate degradation rates respective amounts of five-fold diluted propionic acid (200 g propionic acid per L, 2.7 M) were added to the reactor effluent. Although propionic acid was added it reacted to propionate in the digesters at a pH of around 7 and thus we use the term “propionate” in the following text. Effluent of the municipal biowaste digestion plant of Karlsruhe served for initial inoculation of the reactors.

**Table 1:** Composition of biowaste suspensions.

Parameters	Average Values
Total solids, TS (%)	6.1 ± 0.5
Volatile solids, VS (%)	5.2 ± 0.4
Chemical oxygen demand, COD (g·L^−1^)	94–113
Total Kjeldahl Nitrogen, TKN (g·L^−1^)	2.2 ± 0.2
NH_4_^+^-Nitrogen (g·L^−1^)	0.5 ± 0.1
pH	4.5
Acetate (g·L^−1^)	3.1 ± 0.3
Propionate (g·L^−1^)	2.7 ± 0.3
*n*-Butyrate (g·L^−1^)	1.5 ± 0.2

### 2.2. Reactor Setup, Feeding, and Incubation Conditions

**Figure 1:**
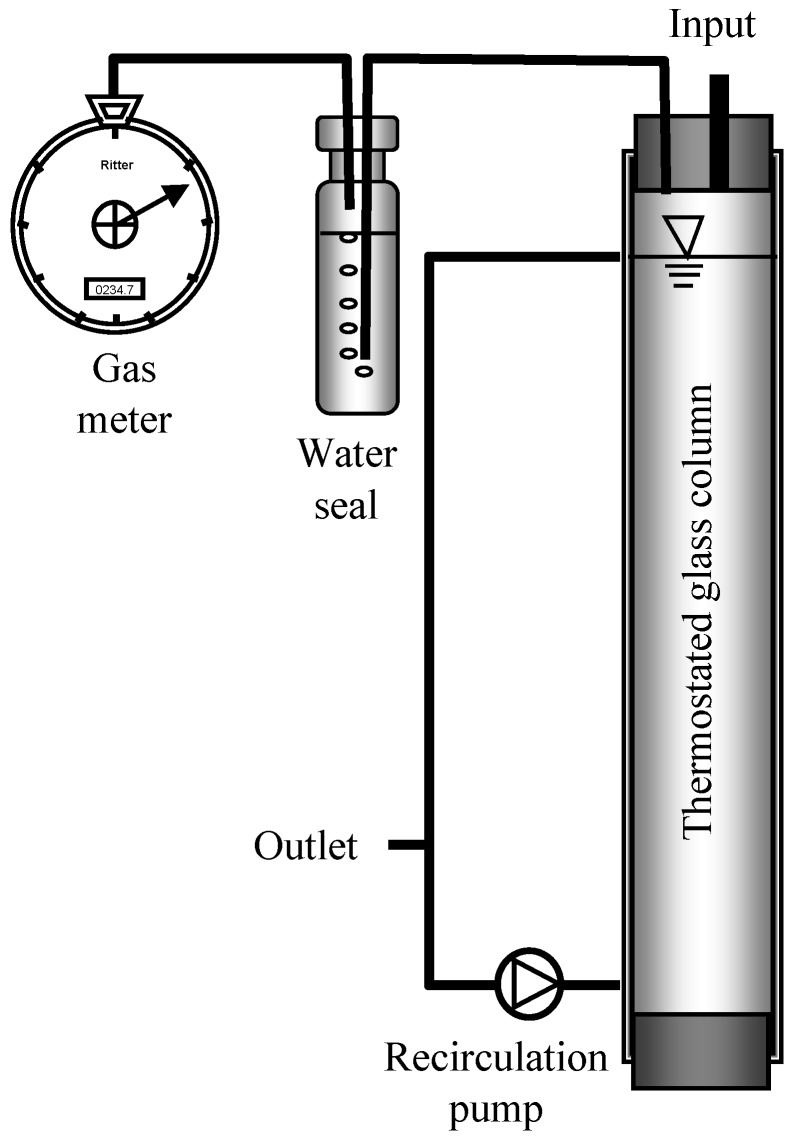
Scheme of reactors.

**Figure 2:**
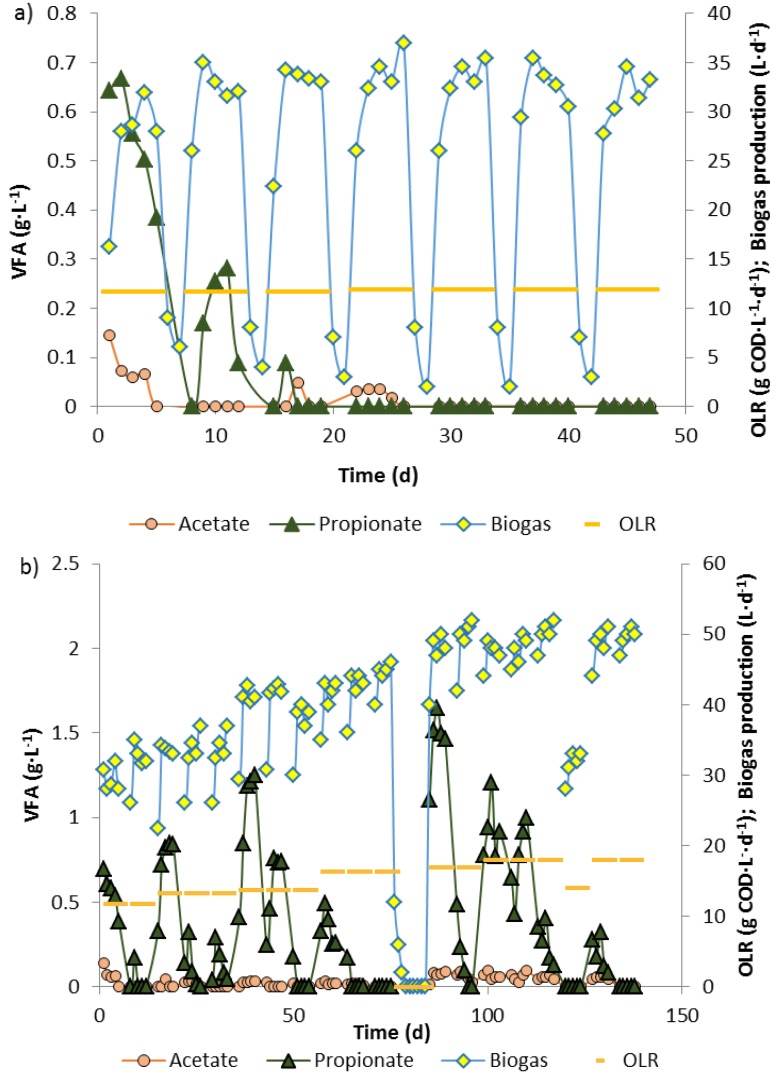
Biogas production and fatty acid levels in a 10 L biowaste reactor 1 after start at an OLR of 12 kg COD_biowaste_·m^−3^·d^−1^ (**a**) and in reactor 2 for increasing organic loading rates up to 18 kg COD·m^−3^·d^−1^, maintained by 12 kg (day 1–55) or 14 kg (new batch biowaste from day 55 onwards) COD_biowaste_·m^−3^·d^−1^ plus respective amounts of propionate (**b**). No feeding between days 75–85 due to maintenance work.

Four cylindrical glass reactors (Figure 1) with a total volume of 10 L and a working volume of 8 L, wrapped with silicon tubing for warm water circulation from a thermostat to maintain 37 °C, were fed from Monday to Friday at 8 a.m. and 6 p.m. with fresh biowaste suspension, replacing 600 mL of digested biowaste. No feed was added on Saturday and Sunday as in the full-scale plant of Karlsruhe. The laboratory reactor 1 was run as a control for 48 days at 12 kg COD·m^−3^·d^−1^ OLR maintained with biowaste suspension batch 1. Reactor 2 was also run with an OLR of 12 kg COD·m^−3^·d^−1^ for 55 days with the same biowaste suspension batch 1, but after 2 weeks the OLR of 12 kg COD_biowaste_·m^−3^·d^−1^ was increased to 13 kg COD·m^−3^·d^−1^ by propionic acid addition. After 55 days the basic OLR with biowaste suspension batch 2 was 14 kg COD_biowaste_^.^m^−3^·d^−1^, which was increased stepwise by addition of respective amounts of propionic acid (addition of 0.7 g propionic acid per L equals an OLR increase of 1 kg COD·m^−3^·d^−1^) to a maximum of 18 kg·m^−3^·d^−1^, as indicated in Table 2 and Figure 2b. During glass repairs (days 75–85, Figure 2b) the reactor content was stored anaerobically but was not fed. Reactor 3 was run with a basic OLR of 3 kg COD·m^−3^·d^−1^, maintained by propionic acid addition all week to keep the propionate-oxidizing bacteria (POB) active. Biowaste suspension was available from Monday to Friday and was added to give an additional OLR of 11 kg COD·m^−3^·d^−1^. Reactor 4 was fed with biowaste suspension from Monday to Friday at an OLR of 11 kg COD·m^−3^·d^−1^ and with propionic acid from Friday night to Monday morning at an OLR of 5 kg COD·m^−3^·d^−1^. With this feeding regime, the same daily gas production as with biowaste, necessary for continuous operation of a generator, was obtained.

For analyses of propionate degradation rates, duplicate assays in serum bottles, containing 40 mL effluent of reactor 2 during operation at different OLRs or of reactor 4 after biowaste or propionate feeding as sources of microorganisms and 800 ± 50 mg L^−1^ propionate, were incubated at 37 °C. Samples were withdrawn with a syringe and the concentration of propionate was determined by gas chromatography. Mean degradation rates were calculated for the exponential degradation phases of propionate in the parallel assays.

**Table 2:** Propionate degradation rates in reactor 2 for biowaste at an OLR of 12/14 kg COD·m^−3^·d^−1^ and after a stepwise increase of the OLR to 18 kg COD·m^−3^·d^−1^ by co-feeding of propionate.

Time (days)	OLR (kg COD·m^−3^·d^−1^)	Propionate Addition (g·L^−1^)	Degradation Rate (mg·L^−1^·h^−1^)
2	12	-	40.4 ^b^
12	12	-	41.4 ^c^
30	12 +1 ^a^	0.7	54.8 ^b^
72	14 + 2.5 ^a^	1.5	70.8 ^b^
94	14 + 3.0 ^a^	1.9	99.9 ^b^
117	14 + 4.0 ^a^	2.5	109.2 ^b^

Biowaste contributed 12 (batch 1 until day 30) or 14 kg COD·m^−3^·d^−1^ (batch 2, after day 30). ^a^ Additional OLR by propionate addition, ^b^ Average propionate degradation rate (±0.3) determined in parallel incubations. ^c^ Average propionate degradation rate of duplicate samples (±0.3) of reactor 1 and reactor 2, as well as from the full-scale biowaste reactor of the city of Karlsruhe.

### 2.3. Analyses

Parameters of the biowaste suspension were determined according to APHA, AWWA, or WEF [18]. Ammonia was quantified with a test kit from Dr. Lange (Berlin, Germany). Acetate, propionate, i- and *n*-buyrate, as well as i- and *n*-valerate, were separated at 160 °C in a Chromosrob C101 Teflon column by gas chromatography with FID detection [4]. Hydrogen, methane, and carbon dioxide (detection limit ≥ 0.05 mmol·L^−1^) were analyzed using a gas chromatograph with a thermal conductivity detector [4]. All values are the mean of at least duplicate analyses. Biogas production was analyzed with Ritter mini gas counters (BlueSens Gas Sensor GmbH, Herten, Germany).

### 2.4. Characterization of the Biowaste Community by Fluorescence in situ Hybridization (FISH)

Samples were withdrawn from the reactors fed with biowaste or biowaste + propionate and treated according to Felchner-Zwirello* et al.* [10]. Parallel samples of 0.1 mL were mixed with 0.3 mL of 4% para-formaldehyde solution [19], incubated at 4 °C for 3 h, and then centrifuged at 15,000 rpm for 5 min in a Microfuge (Eppendorf, Hamburg, Germany). The pellets were washed in phosphate-buffered saline solution (PBS). Samples were frozen at −20 °C in 1 mL 50% ethanol-PBS-solution before further analysis.

For FISH the samples were dried in ethanol, suspended in hybridization buffer, and incubated with gene probes for the domains *Bacteria* and *Archaea*, the orders *Methanomicrobiales* and *Methanosarcinales*, the genera *Methanosaeta*, *Pelotomaculum*, and *Syntrophobacter*, and the species *Smithella propionica*, as described by Li* et al.* [8]. After washing and counterstaining with DAPI, samples were ready for fluorescence microscopy.

### 2.5. Community Density Calculation

Fluorescence-labeled samples were viewed under a Zeiss AxioskopA50 microscope equipped with a mercury HBO 50 UV lamp and an Axiocam camera [8]. Microscopic images were processed with Axiovision 3.1 or DAIME software [20]. From each sample 10 randomly chosen microscopic view fields were photographed using the respective filters for the different fluorescent dyes. Cell numbers represent the un-weighted mean of 10 microscopic view fields of two separate sample preparations. The lowest detection limit was 1.6 × 10^6^ cells·mL^−1^.

## 3. Results

### 3.1. Biowaste Digestion and Co-Digestion Strategies of Propionate

During feeding of an anaerobic digester (reactor 1) with biowaste suspension from Monday to Friday and interruption of feeding on Saturday and Sunday, maintaining an OLR of 12 kg COD·m^−3^·d^−1^, propionate accumulated in the first 3 weeks during the 5 days with biowaste feeding, but was completely degraded during Saturday and Sunday, when no biowaste was supplied (Figure 1a). On this feeding schedule and for this OLR, which both represent a simulation of the full-scale operation of the biowaste digester of the city of Karlsruhe, a steady state was reached 25–27 days after start with a high enough methanogenic activity to completely degrade residual propionate and acetate during the weekend (Figure 2a). When the biowaste/microorganisms in reactor 1 were not fed with fresh biowaste suspension on Saturdays and Sundays biogas production rapidly decreased from average 34 L·d^−1^ from Tuesday to Friday to less than 5 L·d^−1^ until Monday morning. After resuming biowaste feeding, on Mondays biogas generation (Figure 2a, days 8, 15, 22, 29, 36, 43) was much lower than on Tuesday till Friday, indicating metabolic limitations. Only about 35 days after start was the metabolic activity of the community stable and high enough so that after starvation and resuming feeding on Monday only slightly less biogas (28–30 L·d^−1^) was produced than from Tuesday to Friday (31–35 L·d^−1^).

**Figure 3:**
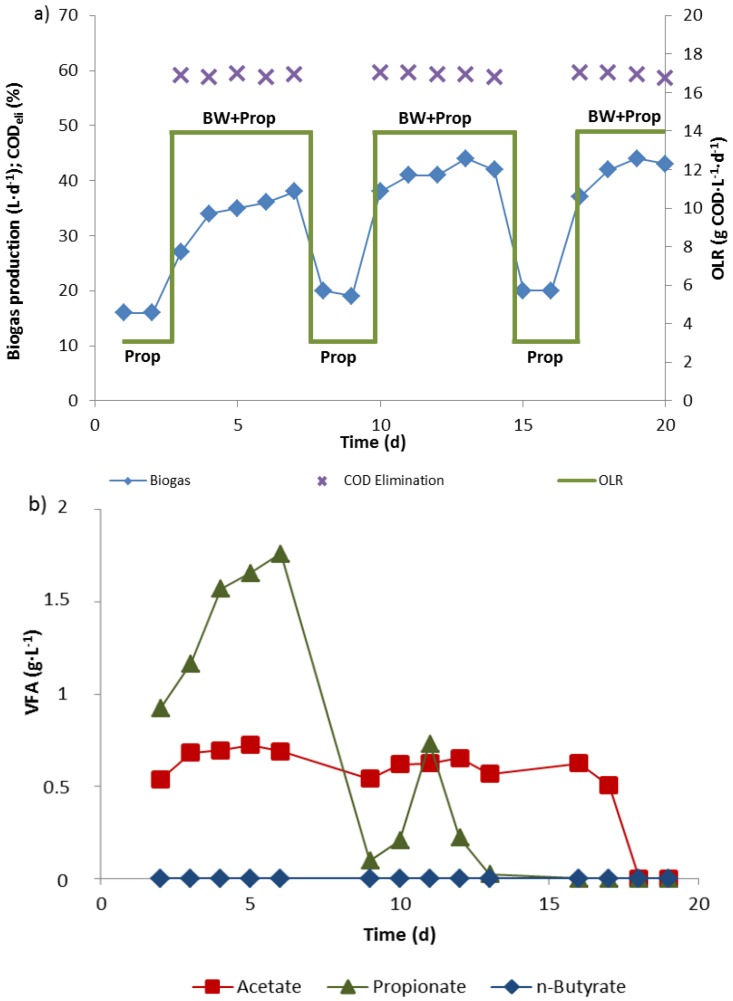
Biogas production (**a**) and fatty acid levels (**b**) in a 10 L biowaste digester (reactor 3) fed constantly with propionate (2.68 mM, 80 mL·d^−1^) at an OLR of 3 kg COD·m^−^^3^·d^−^^1^ and additionally with biowaste from Monday to Friday (1 L·d^−1^) to reach an OLR of 14 kg COD·m^−^^3^·d^−^^1^.

Another reactor (reactor 2) was operated with a basic OLR of 12 (day 1–55) or 14 kg COD_biowaste_·m^−3^·d^−1^ (day 55 onwards) and the high basic load was stepwise increased to 18 kg COD·m^−3^·d^−1^ by co-feeding increasing amounts of propionate (Figure 2b). After every increase of the OLR, during the week high amounts of propionate (higher than the added propionate) accumulated, which were degraded during the starvation periods on weekends. For each stepwise increased OLR, propionate accumulation during the second week of feeding was significantly lower than during the first week and no propionate or only very little propionate accumulated in the third week. With increasing OLR, gas production increased with lower values on Monday than from Tuesday to Friday (Figure 2b, days 0–75), indicating some activity stagnation during the weekends without feeding. During 10 days’ interruption of the feeding for glass repairs (Figure 2b, day 75–85), POB lost much of their metabolic activity. After resuming biowaste + propionate feeding at almost the same OLR as before maintenance, when no propionate was detected in the digester effluent, the highest propionate peak of all was measured (Figure 2b, day 89). Complete regeneration of the propionate degradation activity by POB took more than 40 days. Finally, at the very high OLR of 18 kg COD·m^−3^·d^−1^, maintained with biowaste + propionate feeding for 5 days per week with no feeding on Saturday and Sunday, steady state conditions without residual fatty acids in the effluent were obtained (Figure 2b, days 130–140 and further).

A third reactor (reactor 3) was operated with 3 kg COD_propionate_·m^−3^·d^−1^ as a “background” OLR all week to maintain a steadily high propionate oxidation activity. From Monday to Friday biowaste suspension was additionally supplied to raise the OLR to 14 kg COD·m^−3^·d^−1^ (Figure 3). The minimal biogas production with propionate alone during weekends was 16–18 L·d^−1^. It increased to more than 40 L·d^−1^ when the OLR was increased Monday to Friday by feeding fresh biowaste suspension (Figure 3a). Two weeks after the start, no propionate accumulated in the 10 L reactor during the week anymore, when the OLR was raised from 3 kg COD_propionate_·m^−3^·d^−1^ to 14 kg COD·m^−3^·d^−1^ by biowaste addition. Acetate in the effluent disappeared completely after 3 weeks (Figure 3b).

**Figure 4:**
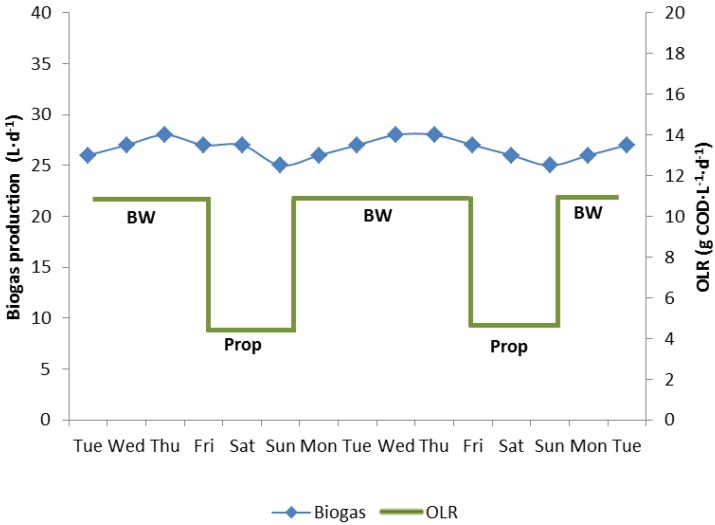
Periodic feeding of biowaste (BW, 1 L·d^−1^) or propionate (Prop, 2.68 M, 120 mL·d^−1^) to maintain an almost constant gas production in the 10 L biowaste reactor 4 over weekends, when no biowaste was available. No fatty acids were detected at any time.

A fourth reactor (reactor 4) was run with biowaste feeding at an OLR of 12 kg COD·m^−3^·d^−1^ from Monday until Friday and with propionate feeding at an OLR of 5 kg COD·m^−3^·d^−1^ from Friday night to Monday morning to maintain a constant gas production 7 days a week (Figure 4a). No propionate or acetate was detected in the digester effluent at any time. Thus the fermentation was stable, representing steady state conditions.

### 3.2. Propionate Degradation Rates

Propionate degradation rates (PDRs) at an OLR of 12 kg COD_biowaste_·m^−3^·d^−1^ in reactor 1 or 2 (Table 2) did not change with time and were between 40.4 and 41.4 mg·L^−1^·d^−1^. The PDRs in reactor 2 with a high basic OLR of 12/14 kg COD_biowaste_·m^−3^·d^−1^ plus increasing amounts of propionate at the final OLR of 18 kg COD·m^−3^·d^−1^ increased to 109.2 mg·L^−1^·d^−1^ (Table 2) and to 100 mg·L^−1^·d^−1^ in only propionate-fed reactor 4. PDRs increased with the addition of propionate (Table 2).

**Figure 5:**
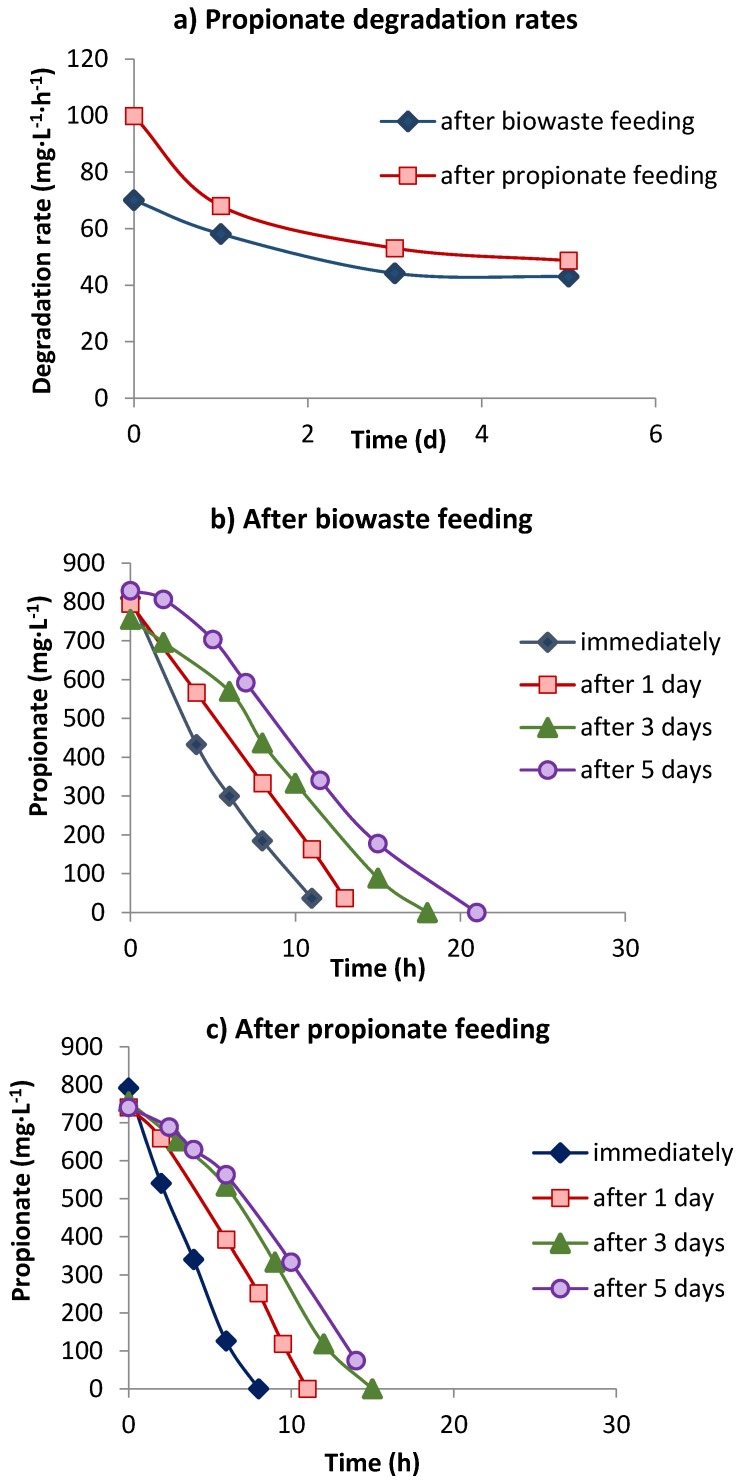
Propionate degradation rates (**a**) and degradation activity immediately following starvation in effluent of reactor 4 or after 1–5 days, after biowaste (**b**) or propionate feeding (**c**).

In reactor 4 with successive feeding of 12 kg COD_biowaste_·m^−3^·d^−1^ for 5 days and 5 kg COD_propionate_·m^−3^·d^−1^for 2 days over the weekend, resulting in an identical daily gas productivity, sludge withdrawn after biowaste feeding had a PDR of 70 mg·L^−1^·h^−1^ (Figure 5a), which was almost double (70 compared to 40 mg·L^−1^·d^−1^) that in the only biowaste-fed reactor 2 (Table 2) at the same loading (Figure 2a; Table 2). Sludge that was withdrawn after 2 days of only propionate feeding had a much higher PDR of 100 mg·L^−1^·h^−1^, compared to the PDR of 109.3 mg·L^−1^·h^−1^ in reactor 2 with 14 kg COD_biowaste_·m^−3^·d^−1^ + 4 kg COD_propionate_·m^−3^·d^−1^. However, during the first day of starvation the PDR decreased much faster in the sludge-fed propionate than in the sludge-fed biowaste (Figure 5a). About 30–50% of the propionate oxidizing activity (POA) was lost during 5 days of starvation, and degradation of propionate in the assays had started already after 3 days’ starvation, after a lag phase of 4–7 h (Figure 5b,c). Propionate degradation was completed much earlier in propionate-fed sludge than in biowaste-fed sludge (Figure 5b,c). While during the first day of starvation POA in the propionate-fed sludge decreased much faster than in the biowaste-fed sludge (30%* versus* 13%), later on the POA in both assays decreased at similar rates (Figure 5a).

### 3.3. Community Changes during Biowaste and Propionate Degradation

**Figure 6:**
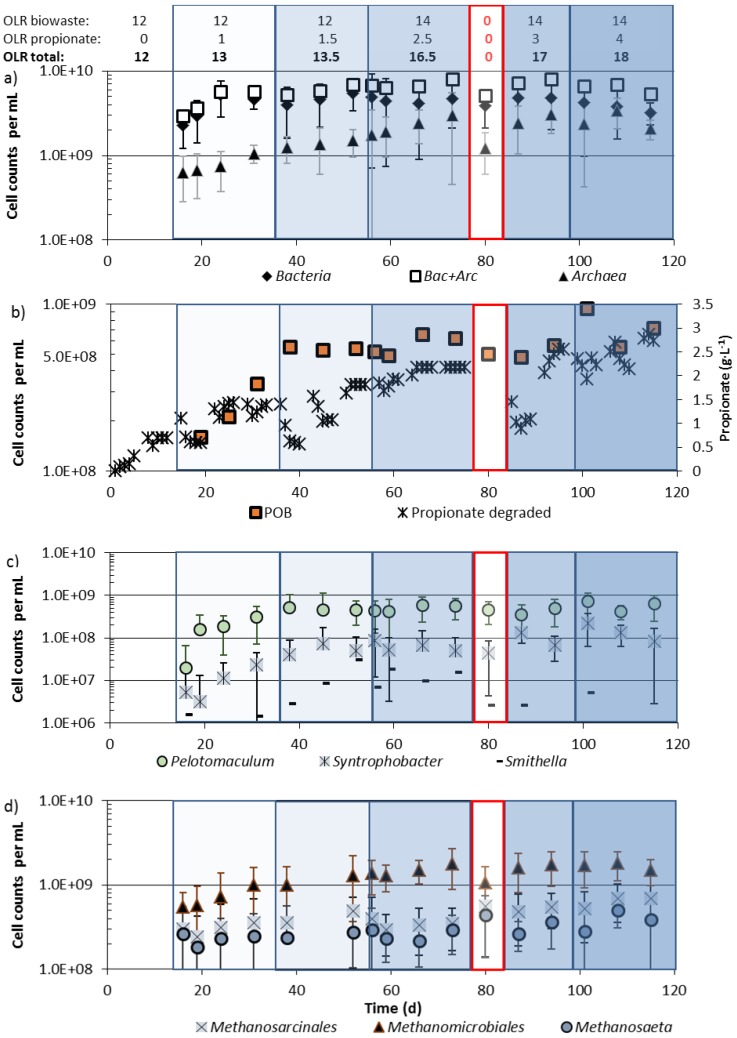
Total *Bacteria* and *Archaea* (**a**), propionate-oxidizing bacteria (POB; **b**, **c**), and methanogenic archaea (**d**) in reactor 2, during feeding of biowaste at an OLR of 12 (day 1–55) or 14 (day 55 onwards) kg COD_biowaste_·m^−3^·d^−1^ and propionate up to 4 kg COD_propionate_·m^−3^·d^−1^. For sample preparation see Section 2.4.

Community changes were investigated in reactor 2, which was fed with biowaste suspension at an OLR of 12 kg COD·m^−3^·d^−1^ until day 55 (batch 1) and 14 kg COD·m^−3^·d^−1^ from day 55 onwards (batch 2) as well as with increasing amounts of propionate, up to a final OLR of 18 kg COD·m^−3^·d^−1^. After only biowaste feeding at an OLR of 12 kg COD·m^−3^·d^−1^, the bacterial community consisted of (2 ± 1.1) × 10^9^ *Bacteria* and (0.6 ± 0.35) × 10^9^ *Archaea* per mL. When the OLR of 12 kg COD_biowaste_·m^−3^·d^−1^ was increased by 1 kg COD_propionate_·m^−3^·d^−1^ the *Bacteria* increased within less than 10 days to their maximum cell density. Highest cell densities of *Archaea* and highest biogas production were, however, reached about 60 days later (Figure 2b and Figure 6a), when up to 2.5 kg COD_propionate_·m^−3^·d^−1^ were supplied in addition to 14 kg COD_biowaste_·m^−3^·d^−1^, presumably due to much slower growth rates of hydrogenotrophic and/or aceticlastic methanogens than of heterotrophic bacteria. More propionate (up to 4 kg COD·m^−3^·d^−1^) in the presence of 14 kg COD_biowaste_·m^−3^·d^−1^ did not lead to further growth and higher community densities of *Bacteria* and *Archaea* (Figure 6a, day 85 onwards).

During revision of reactor 2 (1 week interruption without feeding from day 75–85) *Bacteria* survived almost completely, whereas the *Archaea* apparently suffered from starvation and cell numbers decreased more than 50%, e.g., by cell lysis (Figure 6a). Recovery of the *Archaea* after resuming biowaste + propionate feeding required about 10 days. Addition of propionate to the biowaste reactor resulted in a more than 5-fold increase of the numbers of *Archaea* from 0.6 to 3.4 × 10^9^ per mL (Figure 6a).

During biowaste digestion, less than 10% (1.5 × 10^8^ per mL) of the *Bacteria* were propionate-oxidizing bacteria (POB). When 1–1.5 kg COD_propionate_·m^−3^·d^−1^ were fed in addition to 12 kg COD_biowaste_·m^−3^·d^−1^the community of POB increased more than 3-fold within less than 30 days to at least 5 × 10^8^ per mL (Figure 6b). Most of the POB belonged to the genus *Pelotomaculum*. Only about 10% of the POB were species of *Syntrophobacter* and only about 1% of the POB were species of *Smithella* (Figure 6c). During feeding of 1 or 1.5 kg COD_propionate_·m^−3^·d^−1^ and 12 kg COD_biowaste_·m^−3^·d^−1^, all POB genera increased to their maximum cell density (Figure 6c). If more propionate was co-fed the community density of POB did not increase further, but *Smithella* sp. seemed to be less tolerant to high propionate concentrations or were less competitive against upcoming POB and their numbers decreased, similar to during starvation (Figure 6c, day 80). Highest cell numbers for *Pelotomaculum* were (7.1 ± 4.1) × 10^8^ per mL and for *Syntrophobacter* (2.2 ± 1.6) × 10^8^ per mL (Figure 6c) and members of both genera survived starvation for one week without a decrease of cell numbers.

Within the community of methanogenic archaea, *Methanosarcinales* (acetate-utilizing *Methanosaeta* and *Methanosacina* sp.) as well as *Methanomicrobiales* (H_2_/CO_2_-utilizing methanogens) increased in the first 40 days after propionate co-feeding, when *Methanosarcinales* reached their maximum cell density. The cell density of *Methanomicrobiales* still increased up to day 75 (Figure 6d). If cell densities of *Methanomicrobiales* and *Methanosarcinales* correlated with metabolic activity, and methane production rates of members of both orders were the same, then most biogas released at high OLRs must come from *Methanomicrobiales* since their cell numbers still increased, while the number of *Methansarcinales* stagnated at an OLR of 13.1 kg COD·m^3^·d^−1^ (Figure 6d).

Within the archaeal domain the majority of species were members of the order *Methanomicrobiales*. In the reactor fed with biowaste and propionate, numbers of *Methanomicrobiales* increased from 0.5 to 2 × 10^9^ per mL. The numbers of *Methanomicrobiales* increased more after addition of propionate than those of the *Methanosarcinales*, which were present at much lower cell densities (Figure 6d). In the reactor fed biowaste + propionate (Figure 6d) and in the reactor fed with biowaste alone, the genus *Methanosaeta* (55–84%) represented the majority within the *Methanosarcinales* [6].

## 4. Discussion

### 4.1. Physiological Aspects of Biowaste and/or Propionate Degradation

Under steady state conditions during anaerobic digestion of biowaste and/or propionate, production rates must match with conversion rates to biogas. During stepwise increase of the OLR in the biowaste digester by biowaste or propionate addition acetogenic bacteria and methanogenic archaea or methanogenic archaea, respectively, were limiting factors for quantitative degradation. If the OLR in the biowaste reactor was stepwise increased, after each increase acetate and/or propionate were found in the reactor effluent for a few days and then disappeared (Figure 2) or stabilized at a much lower concentration [2], apparently due to bacterial growth or activity gain. At a high OLR of 16–18 g COD·L^−1^·d^−1^, in the first week propionate concentrations reached 1–1.6 g·L^−1^. With time all propionate disappeared, at the latest during weekends without feeding, as similarly observed by Gallert* et al.* [2]. In the presence of inhibitors, or at high loading close to overload conditions, the capacity of methanogenesis may be exceeded and fatty acids such as propionate or acetate and n-butyrate begin to accumulate. Fatty acids in the effluent indicate incomplete methanogenesis, but some residual propionate in the effluent must not necessarily indicate already a breakdown of biogas production. Pullammappallil* et al.* [21] observed that during phenol inhibition in a glucose-fed anaerobic digester 2.75 g·L^−1^ propionate and about 0.1 g·L^−1^ acetate as well as n-butyrate accumulated, but methanogenesis did not collapse. Amani* et al.* [22] showed that propionate concentrations in an anaerobic digester significantly influenced propionate degradation. When the propionate concentration was 3 g·L^−1^, 17% less propionate was removed than at a lower concentration of only 1.5 g·L^−^^1^. After addition of propionic acid and during stepwise increase of the OLR with biowaste, propionate concentrations in the effluent of our biowaste reactors sharply increased during the first days, but the propionate was completely degraded during weekends without feeding. POB and propionate degradation activity increased with time and almost no propionate accumulated in the third week of propionate feeding at the same OLR. The maximum propionate accumulation was 1.6 g·L^−1^ in reactor effluent, when 1.9 g·L^−1^ propionate was added with the feed. The effluent propionate concentration represented external addition plus internal production by fermenting bacteria minus degradation by syntrophic interaction of POB and methanogens. Biogas production increased with time and the pH in the digester remained stable throughout the stepwise increase of the OLR up to 18 kg COD·m^−3^·d^−1^ with biowaste and propionate co-feeding. There was a strong Pearson correlation of added propionate and biogas production of 0.86 until day 90 for an OLR of 14 kg COD_biowaste_ + 2.5 kg COD_propionate_·m^−3^·d^−1^. When more propionate was added (Figure 2b, day 85–140), biogas production no longer increased, indicating not only a reduced biowaste methanation but presumably also reduced acidification, since no fatty acids were accumulating and the previously accumulated propionate was apparently degraded over time. Thus the addition of 2.5 kg COD_propionate_·m^−3^·d^−1^ together with 14 kg COD_biowaste_·m^−3^·d^−1^ was the maximum amount for co-digestion at maximal degradation efficiency and maximal biogas production.

In Karlsruhe, Germany, source-sorted municipal biowaste is collected Monday to Friday and a biowaste suspension for anaerobic digestion prepared on the same day. Since the storage capacity for the biowaste suspension is limited, the suspension is digested on the same day. Storage of freshly collected biowaste from Friday until Monday morning is avoided to prevent spoilage by biocide-forming *Phycomycetes* which might inhibit methanogenesis. No storage tank for digested suspension is available, requiring solid separation by centrifugation immediately after removal of digested biowaste suspension from the reactor. Thus, a sufficient biogas amount for operation of a gas engine and an electricity generator is available during working days and only very little biogas is produced during weekends without fresh feed. To overcome this restriction, removal of biowaste suspension to the minimum filling level of the reactor on Friday night and automatic addition of highly concentrated pre-acidified liquid wastes during the weekend up to the maximum filling level on Monday morning would allow a constant gas production, as shown here with propionic acid as a “model liquid waste.” Nayono* et al.* [17,23] reported already that fine-particles containing suspensions of highly concentrated food waste or press water from biowaste would be suitable substrates for automatic pump feeding of biowaste digesters during weekends. Such substrates are much more homogeneous than biowaste and would allow plant operation in the absence of inspecting staff. Acidified liquid substrates with high concentrations of fatty acids such as acetate, propionate, or n-butyrate might be used for automatic feeding of a biowaste reactor during phases when no biowaste is available, since these volatile fatty acids could be rapidly degraded without a significant lag-phase immediately after biowaste feeding. A rapid degradation of volatile fatty acids containing liquid wastes is possible even when no fatty acids remain in the reactor effluent during the week, as shown in this study. Since fatty acids such as propionate in acidified substrates are 100% degradable in syntrophic associations of acetogenic bacteria and methanogenic archaea, for a similar biogas production a much lower OLR is necessary during feeding of highly concentrated acidified substrates than during feeding of complex biowaste suspensions, which are only degradable to an extent of 50–70% [17,23].

### 4.2. Community Shifts during Propionate Degradation

When the OLR of reactor 2 was increased from 12 kg COD·m^−3^·d^−1^ to 13 kg COD·m^−3^·d^−1^ by propionate addition, the community density of *Bacteria* reached its maximum within a few days and then remained stable, even when the OLR was further increased with propionate addition, whereas the *Archaea* grew much more slowly and cell numbers increased only over a wide range of increasing OLRs (Figure 6a). This led to changing proportions of *Bacteria* and *Archaea* in the digester. At a propionate contribution of 1 kg COD·m^−3^·d^−1^ to the total OLR of 13 kg COD·m^−3^·d^−1^ in the biowaste reactor, the percentage of *Archaea* within the total community was 18% (Figure 6a, day 32). When propionate contributed 4 kg COD·m^−3^·d^−1^ to the total OLR of 18 kg COD·m^−3^·d^−1^, the proportion of *Archaea* increased to 40% of total prokaryotes (Figure 6a, day 100–120). This was double the highest report by McMahon* et al.* [1], who fed their digester with a simulated synthetic organic fraction of municipal solid waste and reported that 21–23% of total prokaryotes were *Archaea*. Wang* et al.* [24] observed that in a digester fed with pre-treated food waste the proportion of *Archaea* contributed 12–18% to the total number of prokaryotes. Angenent* et al.* [25] digested swine manure and calculated a proportion of 15% *Archaea* within the total prokaryotes. A very high proportion of 64% *Archaea* within the total prokaryotes in a continuously operated, solely propionate fed chemostat can be calculated from the report of Shigematsu* et al.* [12] for a dilution rate of 0.3 h^−1^, indicating the selective enrichment of methanogens and presumably POB. As biowaste is a complex substrate there is a need of a larger spectrum of fermenting *Bacteria* than in digesters with propionate feeding, where only POB and eventually acetate-oxidizing bacteria (AOB) and H_2_/CO_2_, as well as acetate-utilizing methanogens, are required. Therefore, it seems plausible that the proportion of *Archaea* increased with an increasing OLR by propionic acid addition into the reactor.

A strong positive correlation (Pearson correlation 0.86) was found between numbers of *Methanomicrobiales* and biogas production. A supply of more propionate resulted in more biogas and in higher numbers of *Methanomicrobiales*, whereas the numbers of *Methanosarcinales* did not correlate well with biogas production or the higher concentration of propionate. This could mean that most of the methane derived from propionate degradation was generated by the hydrogenotrophic *Methanomicrobiales* community in conjunction with acetate-oxidizing bacteria (AOB) instead of *Methanosarcinales.* Since no acetate was accumulating in the reactor effluent and numbers of acetate-fermenting *Methanosarcinales* remained almost constant at increasing propionate OLRs, AOB may have been active, delivering CO_2_ and H_2_ for the *Methanomicrobiales* community. *Methanosarcinales* in the biowaste/propionate fed reactor stagnated for about 70 days (Figure 6d) and most of the acetate from propionate oxidation presumably was oxidized by AOB. An interaction of AOB and hydrogenotrophic methanogens for acetate cleavage to CO_2_ and hydrogen was already documented by Hori* et al.* [16]. It was reported that e.g., the number of *Methanoculleus*, a genus of *Methanomicrobiale*s, correlated positively with the number of *Pelotomaculum* sp. (Pearson correlation 0.98) [14]. In the present study *Methanoculleus* sp. were not specially enumerated, but a positive correlation between *Pelotomaculum* and the number of *Methanomicrobiales* (Pearson correlation 0.79, [6]) was seen, which was in accordance with data from earlier work with the same source of biowaste [7,26]. There are, however, also examples of both high numbers of *Pelotomaculum* sp. and low numbers of *Methanomicrobiales*, which might indicate a dominance of aceticlastic methanogens [6]. A syntrophic association of *Pelotomaculum* sp., AOB, and members of *Methanomicrobiales* is another possibility but not a “conditio sine qua non” for growth on propionate, as reported by Moertelmaier* et al.* [7].

At relatively stable methane production (Figure 2b, days 85–90, after maintenance), cell numbers of *Bacteria* and of POB recovered, although propionate accumulated (formation > degradation). Cell numbers of the three genera of POB increased at an OLR of 17–18 kg COD·m^−3^·d^−1^ (Figure 6c). The maximum propionate degradation rate was around 2.6 g·L^−1^·d^−1^ (calculated from data of Table 2) at a daily addition of 2.5 g·L^−1^ propionic acid into the reactor (contributing 4 kg CSB·m^−3^·d^−1^ to the OLR). Thus, only little propionate could have been generated from biowaste. When the reactor was fed with biowaste alone, up to 1.7 g·L^−1^ propionate was excreted. Propionate production was apparently suppressed during biowaste plus propionate feeding. Co-digestion of propionate seems to be a way to stabilize and increase the number of POB. Compared with the POB numbers in investigations with biowaste alone, the numbers of POB in digesters that were fed with biowaste + propionate were slightly higher: After the start of the full-scale biowaste digester of Karlsruhe 2–3 × 10^8^ POB per mL were found in reactor effluent [6] and during co-digestion of biowaste with wheat and rye bread at high concentrations of propionate 2–5 × 10^8^ POB per mL were detected [26], whereas 5–6 × 10^8^ POB per mL were found after biowaste/propionate co-digestion (Figure 6c).

When propionate was fed the most numerous POB were *Pelotomaculum* sp. ((5.1 ± 1.6) × 10^8^ mL^−1^), followed by significantly less *Syntrophobacter* sp. ((8.4 ± 0.3) × 10^7^ mL^−1^) and *Smithella* sp. ((1.1 ± 0.9) × 10^7^ mL^−1^, Figure 6c). *Pelotomaculum* sp. and *Syntrophobacter* sp. began to grow when co-fermentation of 1 kg CSB·m^−3^·d^−1^ of propionate in the presence of 12 kg·m^−3^·d^−1^ biowaste was started, whereas *Smithella* sp. began to grow only when 1.5 kg propionate·m^−3^·d^−1^ were present and then approached numbers of *Syntrophobacter* sp. However, at higher OLR *Smithella* sp. decreased again to much lower final cell numbers (<10^7^ mL^−1^) than *Pelotomaculum* sp. or *Syntrophobacter* sp. (>10^8^ mL^−1^; Figure 6c, days 90–120). These results indicate that *Smithella* sp. are not able to compete with the dominant *Pelotomaculum* sp. and with *Syntrophobacter* sp. at very low or very high propionate levels.

## 5. Conclusions

Propionic acid as a model substrate for highly acidified organic liquids could immediately substitute for biowaste suspensions in a biowaste reactor. Addition of little propionate to biowaste suspensions improved the propionate oxidation activity in a biowaste reactor at increasing OLR. An almost constant daily biogas production could be maintained during manual feeding of a biowaste suspension twice a day from Monday to Friday (regular biowaste collection period) and continuous pump feeding of propionic acid as a concentrated model substrate for acidified liquid wastes on Saturday and Sunday, when no biowaste is collected. Propionate degradation rates (PDRs) were much higher after propionate feeding than after biowaste feeding. During 5 days’ starvation the PDR decreased by 50%, but remained higher in biowaste suspensions after propionate feeding than after biowaste feeding.

The total number of bacteria including POB increased with little propionate feeding to its maximum within a few days, while the archaeal community increased slowly for about 60 days. In the biowaste-fed reactors, members of the genus *Pelotomaculum* were the abundant POB. Members of the genera *Syntrophobacter* and *Smithella* were also present, but at much lower numbers. POB reach their maximum cell numbers 20–40 days after the start. Very low numbers of *Smithella* sp. were detected under starvation conditions or at high concentrations of propionate. Numbers of acetate-utilizing methanogens increased only slowly but not proportionally to maximal cell numbers of *Bacteria*, whereas cell numbers of *Methanomicrobiales* increased faster and proportionally to numbers of *Bacteria* with increasing propionate load and reached a much higher cell density after about 70 days.
